# Microbial Diversity in Cerrado Biome (Neotropical Savanna) Soils

**DOI:** 10.1371/journal.pone.0148785

**Published:** 2016-02-05

**Authors:** Alinne Pereira de Castro, Maria Regina Silveira Sartori da Silva, Betania Ferraz Quirino, Mercedes Maria da Cunha Bustamante, Ricardo Henrique Krüger

**Affiliations:** 1 Enzymology Laboratory, Departamento de Biologia Celular, Universidade de Brasilia, Brasília, DF, Brazil; 2 Universidade Católica Dom Bosco, Biotechnology Program, Campo Grande, MS, Brazil; 3 Laboratório de Ecologia de Ecossistemas, Departamento de Ecologia, Instituto de Ciências Biológicas, Universidade de Brasília, Brasília, DF, Brazil; 4 Embrapa-Agroenergy, Brasília, DF, Brazil; 5 Genomic Sciences and Biotechnology Program, Universidade Católica de Brasília, Brasília, DF, Brazil; Universidade Federal do Rio de Janeiro, BRAZIL

## Abstract

The Cerrado, the largest savanna region in South America, is located in central Brazil. Cerrado physiognomies, which range from savanna grasslands to forest formations, combined with the highly weathered, acidic clay Cerrado soils form a unique ecoregion. In this study, high-throughput sequencing of ribosomal RNA genes was combined with shotgun metagenomic analysis to explore the taxonomic composition and potential functions of soil microbial communities in four different vegetation physiognomies during both dry and rainy seasons. Our results showed that changes in bacterial, archaeal, and fungal community structures in *cerrado denso*, cerrado *sensu stricto*, *campo sujo*, and gallery forest soils strongly correlated with seasonal patterns of soil water uptake. The relative abundance of AD3, WPS-2, Planctomycetes, Thermoprotei, and Glomeromycota typically decreased in the rainy season, whereas the relative abundance of Proteobacteria and Ascomycota increased. In addition, analysis of shotgun metagenomic data revealed a significant increase in the relative abundance of genes associated with iron acquisition and metabolism, dormancy, and sporulation during the dry season, and an increase in the relative abundance of genes related to respiration and DNA and protein metabolism during the rainy season. These gene functional categories are associated with adaptation to water stress. Our results further the understanding of how tropical savanna soil microbial communities may be influenced by vegetation covering and temporal variations in soil moisture.

## Introduction

Despite the considerable biodiversity found in Brazil, only recently have efforts been made to describe microbial diversity in the different Brazilian biomes. To better understand patterns of microbial distribution, an initiative has brought together leading microbial diversity studies conducted in various Brazilian biomes during the last 5 years [1], which has provided the information needed for *in silico* analysis of 16S ribosomal RNA (rRNA) genes. Previous studies have shown that some Brazilian soils support more complex microbial communities than others, with an unexplored genetic diversity [2–5]. The soils of the Cerrado biome have been the principal focus of a number of culture-independent analyses of microbial diversity [2, 6–8].

The Cerrado, which is considered the largest savanna region in South America, is located in the central Brazil, bordered by Amazonian forests to the northwest and the Atlantic coastal forest to the southeast [9]. The Cerrado biome is thought to have existed even before the final separation of the South American and African continents. Its physiognomies, which vary from savanna grasslands to forest formations, in combination with its soil conditions (weathered, acidic, with high clay content) form a unique ecoregion [9]. The Cerrado biome offers a home to a wide range of plants, animals, and microorganisms, making it one of the 25 most vital terrestrial biodiversity hotspots described by Myers et al. [10]. However, its true diversity is unclear, as estimates vary considerably among studies. Despite its importance, the cerrado biome has suffered degradation for decades due to human activity and land use changes. The most drastic impact to the biome was the construction of the new capital Brasília in 1960 in the center of the Brazilian Cerrado and the subsequent 50-fold increase in population in this region.

Although many studies have shown that alterations in environmental conditions are important drivers of changes in soil microbial diversity, this has been poorly documented in the Cerrado biome. In particular, detailed studies are needed to understand how soil microbial community structure and function in the Cerrado vary across terrestrial environments, and how soil microbial communities are influenced by the temporal variations in soil moisture associated with vegetation cover. For this reason, this study investigated the bacterial, archaeal, and fungal associations in Cerrado soils within four different vegetation physiognomies using high-throughput DNA sequencing of ribosomal marker sequences in combination with shotgun metagenomic analysis. Revealing diversity at the genetic level will be fundamental to furthering our current understanding of microbial interactions and the potential consequences of future climate change in terrestrial ecosystems.

## Material and Methods

### Sample collection

The study site was located in the Brazilian Institute of Geography and Statistics (IBGE) Ecological Reserve, a protected area in the Federal District of Brazil. Soil samples were obtained from four different vegetation physiognomies: *cerrado denso* (dense tree layer ranging from 5 to 8 m in height) (15° 56' 43.1''S, 47° 51' 26.0''W), cerrado *sensu stricto* (continuous grass layer and a woody layer of trees and shrubs varying in cover between 10% and 60%) (15° 57' 02.4''S, 47° 5' 32.1''W), *campo sujo* (open savanna with widely scattered small trees and shrubs) (15° 56' 54.6''S, 47° 52' 11.7''W), and gallery forest (forest formation along a water course) (15° 57' 06.0''S, 47° 53' 18.7''W).

Soil sampling methods and physicochemical analyses of these habitats were previously described [2]. In the dry season (September 2010) and rainy season (February 2011), three replicate soil samples were obtained from each of the four vegetation physiognomies (24 samples total). Gravimetric water content was calculated based on soil weight before and after the samples were oven-dried at 105°C for 72 h. Physicochemical properties of the soils were determined using samples obtained in the dry season (S1 Table).

### High-throughput DNA sequencing of ribosomal RNA genes

Microbial DNA was extracted from 0.5 g soil from each of the 24 samples using the PowerSoil DNA Isolation Kit (MO BIO Laboratories, Carlsbad, CA, USA) according to the manufacturer’s instructions. The triplicate DNA samples were not pooled.

Bacterial 16S rRNA genes were amplified with the primer pair 787F/1492R [11], archaeal 16S rRNA genes were amplified with the primer pair 751F/UA1406R [12], and fungal 18S rRNA genes were amplified with the primer pair EF4F/Fung5R [13] using a previously described polymerase chain reaction (PCR) protocol [2]. The amplicons were purified using the QIAquick PCR Purification Kit (QIAGEN, Chatsworth, CA) and sequenced using the GS FLX+ Titanium system (454 Life Sciences Corporation, Branford, CT, USA).

### rRNA gene analysis

The rRNA gene analyses were performed using Quantitative Insights Into Microbial Ecology (QIIME) software, version 1.6.0-dev [14]. Briefly, adaptor sequences were trimmed from raw data, with 98% or more of the bases demonstrating a Phred quality score of 30. Sequences were binned into individual sample collections based on barcode sequence tags, which were then trimmed. The resulting files were denoised using the PyroNoise algorithm. Sequences less than 180 bp in length were deleted, and the rest were clustered into species-level operational taxonomic units (OTUs) at 97% sequence similarity using an open-reference OTU picking protocol. Taxonomic assignment was carried out by alignment with the Greengenes database using the Uclust algorithm.

The alpha and beta diversity of the microbial communities were determined. Significant differences between groups were evaluated by analysis of similarities (ANOSIM) [15], and the partial Mantel test was used to evaluate relationships between level of precipitation and microbial community composition. Groups were compared by Fisher’s exact test (confidence intervals with nominal coverage of 95%), followed by the Bonferroni correction using Statistical Analysis of Metagenomic Profiles software, version 2.0.0 [16]. All sequences files are available from the GenBank database Submission BioProject ID: PRJNA298258.

### Shotgun metagenomic analyses

Environmental DNA was extracted from each soil sample using the FastDNA^®^ SPIN Kit for Soil (MP Biomedicals, Santa Ana, CA, USA) and the FastPrep^®^ sample preparation system (MP Biomedicals) according to the manufacturer’s instructions. The 24 DNA samples (1.0 mg per sample) were prepared for sequencing by nebulization, followed by tagging using the GS FLX Titanium Rapid Library MID Adapters Kit (454 Life Sciences).

The metagenomic analysis was conducted using the Metagenomics Rapid Annotation using Subsystem Technology (MG-RAST) server, version 3.2 [17]. Short and low-quality sequences with ambiguous bases (multiple internal Ns) were removed before analysis. Functional analyses were performed using the SEED database (maximum e-value cutoff 1e-10, minimum percent identity cutoff 60%, and minimum alignment length cutoff 50 bp). Groups were compared by Fisher’s exact test (confidence intervals with nominal coverage of 95%), followed by the Bonferroni correction for multiple comparisons using the Statistical Analysis of Metagenomic Profiles software, version 2.0.0. All sequences files are available from the GenBank database Submission BioProject ID: PRJNA298258

## Results

### Characterization of microbial communities by ribosomal gene analysis

To analyze soil microbial communities during the dry season, soil samples were collected from four Cerrado vegetation physiognomies (*cerrado denso*, cerrado *sensu stricto*, *campo sujo*, and gallery forest) in September, after the study site had experienced more than 100 consecutive days without rain. The dry season ended in October, when the study areas received approximately 200 mm precipitation. To analyze soils during the rainy season, samples were collected in February (S1A Fig). As shown in S1B Fig, the gravimetric water content of soils collected during the dry season differed considerably among the four study areas.

From these soil samples a total of 403,568 high-quality bacterial sequences, 27,066 high-quality archaeal sequences, and 35, 979 high-quality fungal sequences were obtained. For all downstream analyses, the samples were rarefied to the smallest number of reads for each domain (bacteria, archaea, or fungi) to correct for differences in sequencing depth.

### Compositions of bacterial, archaeal, and fungal communities in the different vegetation physiognomies were associated with temporal variations of soil moisture

**Fig 1:**
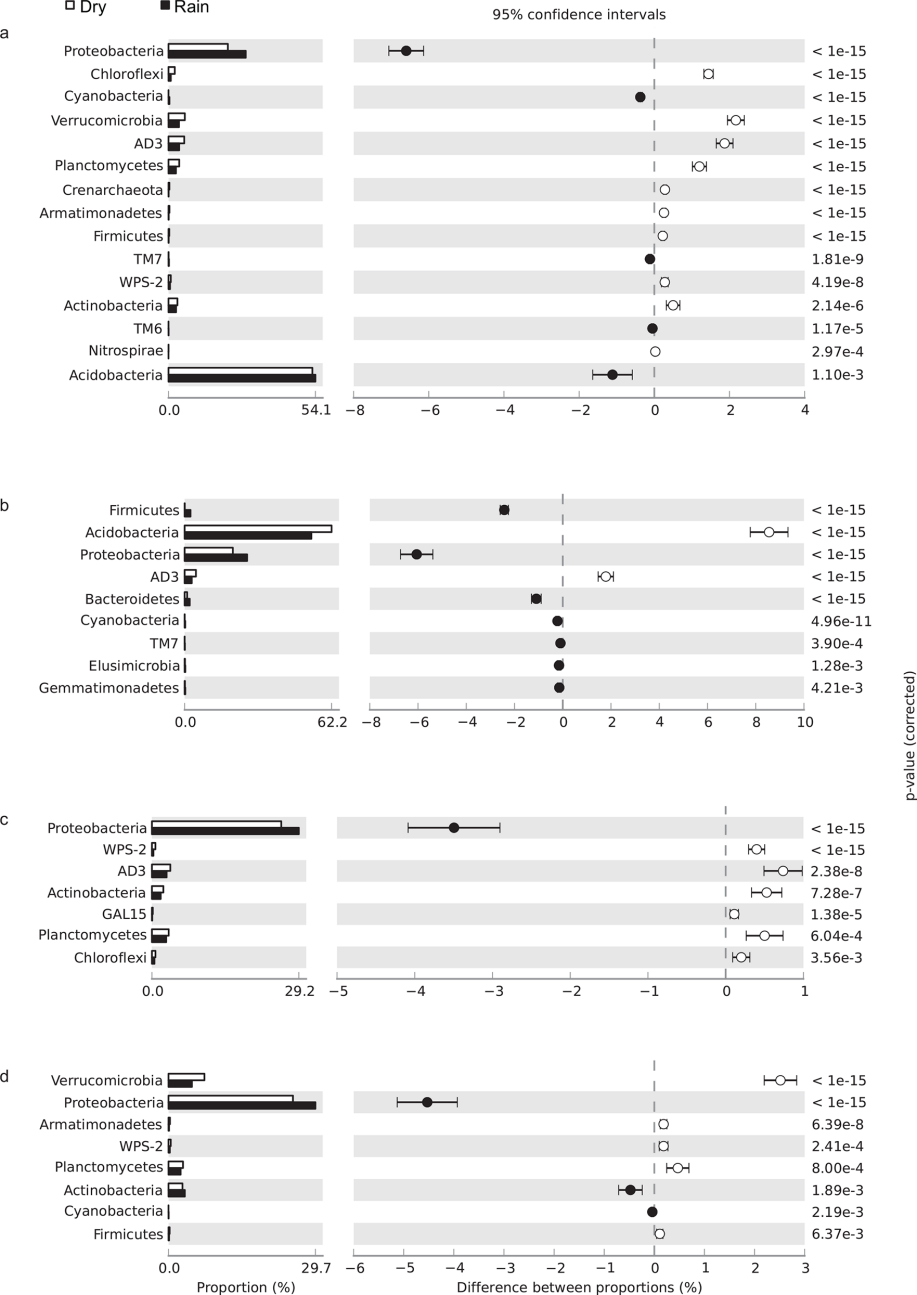
Relative abundance of bacterial phyla in (a) *cerrado denso*, (b) *campo sujo*, (c) cerrado *sensu stricto*, and (d) gallery forest soils, showing differences between the dry season (white bar) and rainy season (black bar); P < 0.05 was considered significant.

The relative abundance of certain phyla in soils of the four vegetation physiognomies varied significantly between wet and dry seasons. For most physiognomies, the relative abundances of phyla AD3, WPS-2, Planctomycetes, Verrucomicrobia, and Chloroflexi decreased during the rainy season, whereas the relative abundance of Proteobacteria increased (Fig 1).

**Fig 2:**
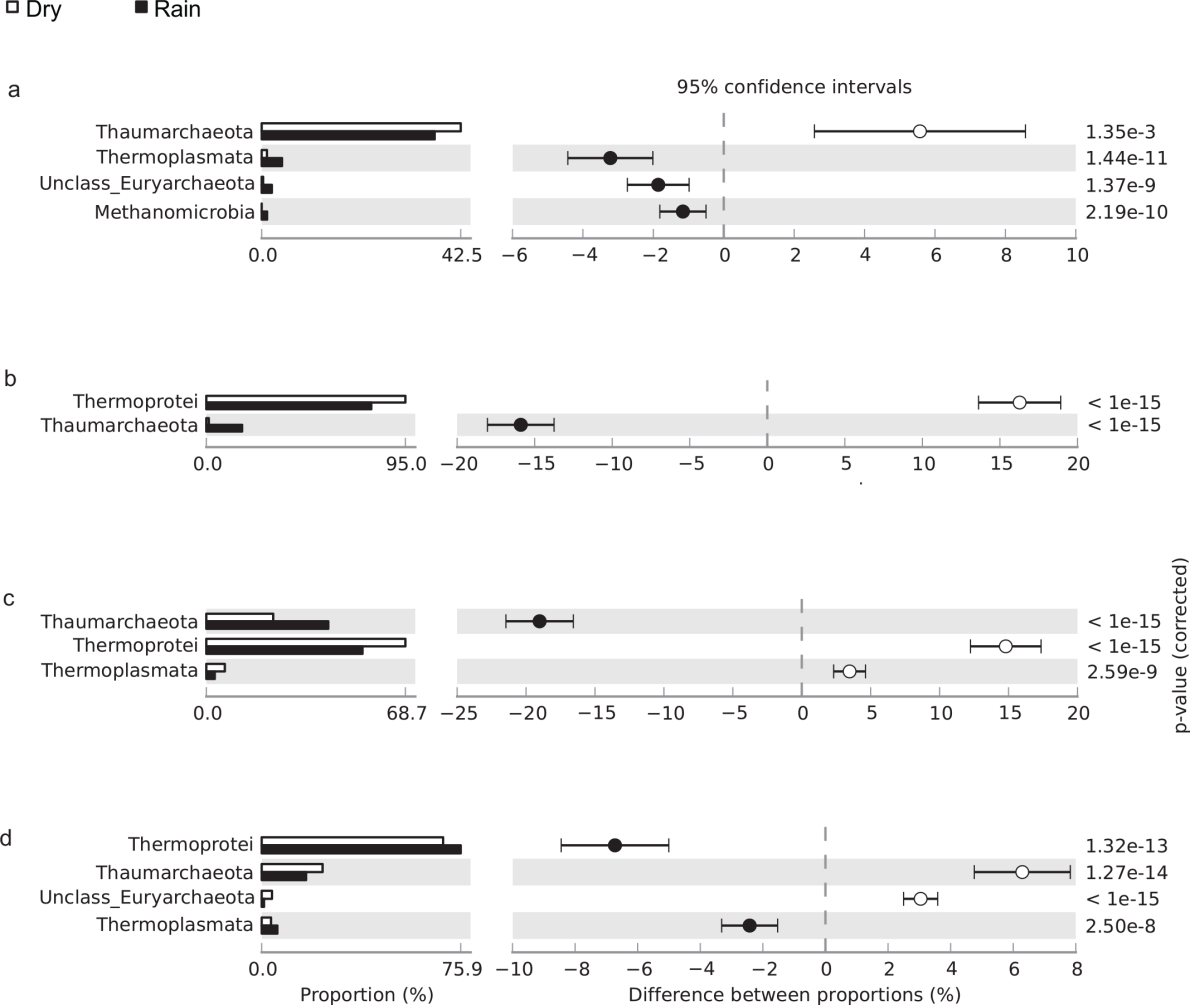
Relative abundance of archaeal phyla and classes in (a) *cerrado denso*, (b) *campo sujo*, (c) cerrado *sensu stricto*, and (d) gallery forest soils, showing differences between the dry season (white bar) and rainy season (black bar); P < 0.05 was considered significant.

In the archaeal community, Crenarchaeota was the most abundant phylum, with Thermoprotei the predominant class detected in all soil samples except those obtained from the *cerrado denso* (Fig 2). The relative abundance of Thermoprotei was higher during the dry season in *campo sujo* and cerrado *sensu stricto* soils but was higher during the rainy season in gallery forest soil (Fig 2).

Sequences affiliated with Euryarchaeota, including Thermoplasmata, Methanobacteria, and Methanomicrobia, were detected in soils of all physiognomies under both dry and rainy conditions but were present in low numbers (data not shown). In contrast, sequences belonging to candidate phyla Korarchaeota and Nanoarchaeota were not detected.

**Fig 3:**
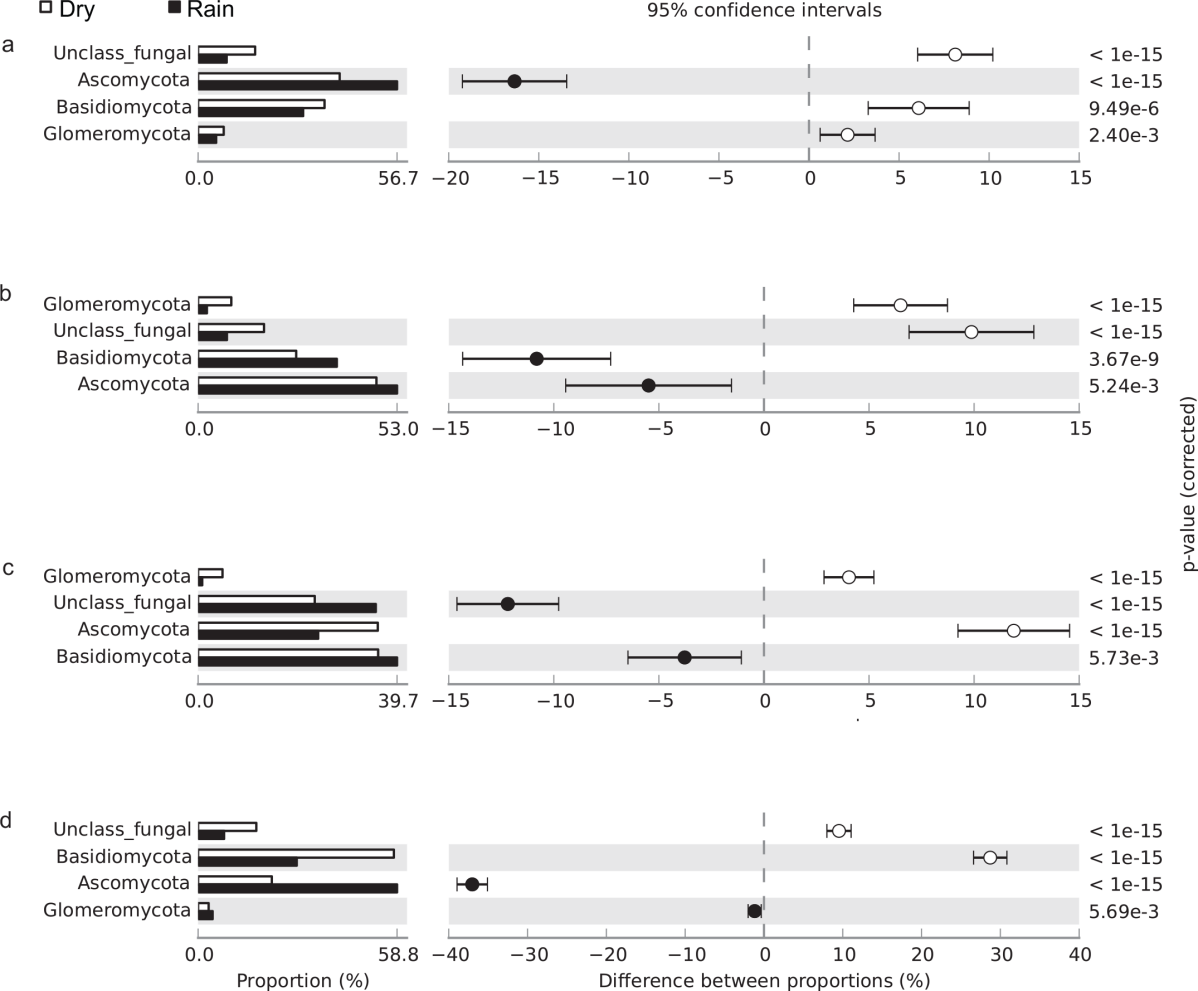
Relative abundance of fungal phyla in (a) *cerrado denso*, (b) *campo sujo*, (c) cerrado *sensu stricto*, and (d) gallery forest soils, showing differences between the dry season (white bar) and rainy season (black bar); P < 0.05 was considered significant.

Fungal community structure also differed significantly between seasons (Fig 3). During the rainy season the relative abundance of Ascomycota was significantly higher in the *cerrado denso*, *campo sujo*, and gallery forest soils but lower in the cerrado *sensu stricto* soil. In contrast, the relative abundance of Glomeromycota significantly decreased during the rainy season in the *cerrado denso*, *campo sujo*, and cerrado *sensu stricto* sites. In addition, the relative abundance of unclassified fungal sequences was lower during the rainy season in *cerrado denso*, *campo sujo*, and gallery forest soils.

### β-Diversity patterns

**Fig 4:**
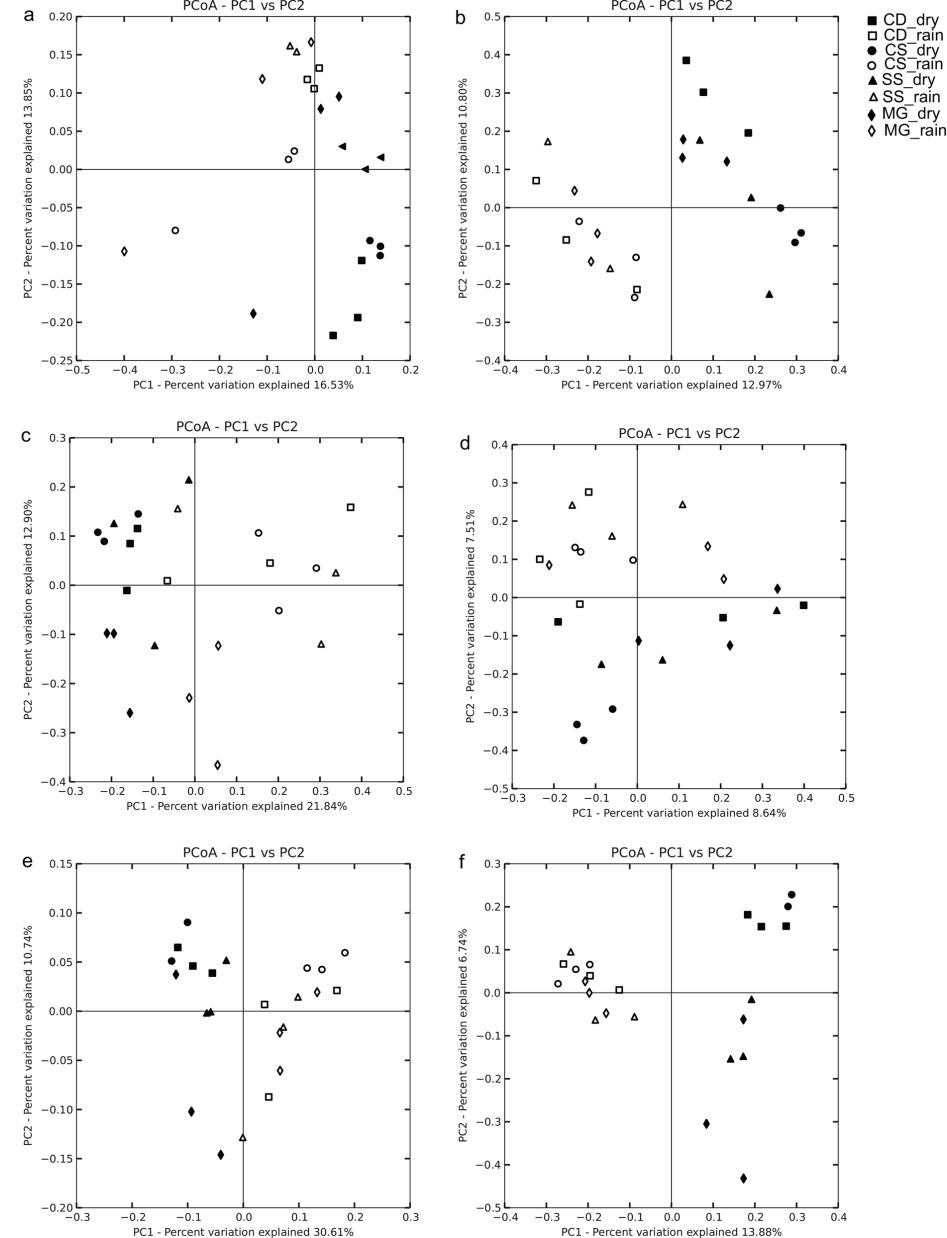
Principal coordinates analysis (PCoA) plots were generated for bacterial, fungal, and archaeal communities to visualize differences between the dry and rainy season and the four Cerrado physiognomies. Analysis of soil bacterial community structure based on (a) unweighted UniFrac distance and (b) Canberra distance; soil archaeal community structure based on (c) unweighted UniFrac distance and (d) Canberra distance; and soil fungal community structure based on (e) unweighted UniFrac distance and (f) Canberra distance. Markers represent replicate soil samples collected during the dry and rainy season in *cerrado denso* (CD, square), *campo sujo* (CS, circle), cerrado *sensu stricto* (SS, triangle), and gallery forest (MG, diamond) soils.

Differences in bacterial, archaeal, and fungal community composition between rainy and dry seasons were demonstrated by both measures of phylogenetic distance, followed by ANOSIM (Fig 4). Results of the Mantel test for pairs of distance matrices (999 permutations for each test) revealed a strong correlation in the unweighted UniFrac measure (Mantel *r* 0.672, 0.448, and 0.495 for bacterial, archaeal, and fungal communities, respectively; *P*< 0.001 in all cases).

**Fig 5:**
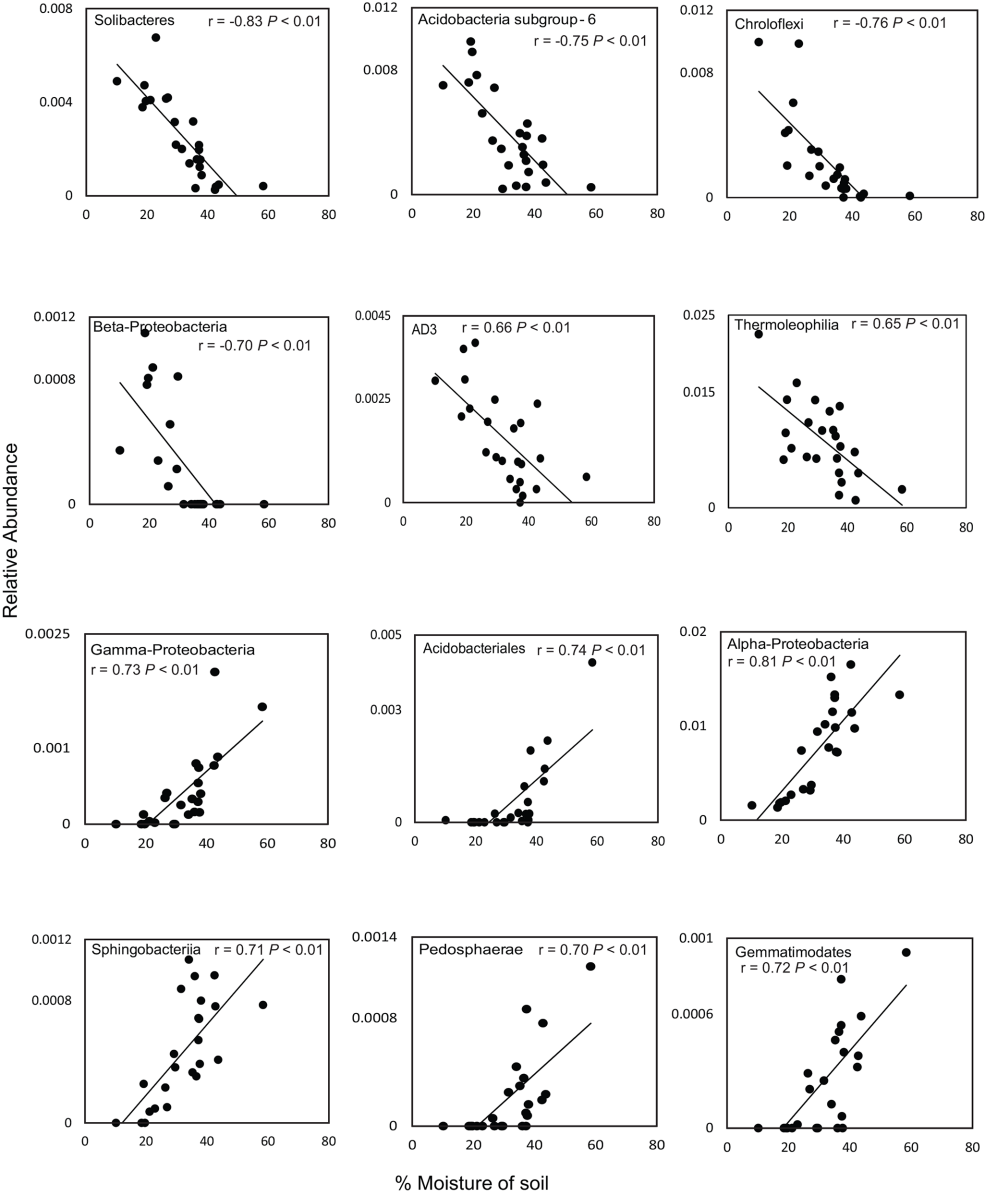
Correlations between soil moisture content and relative abundance of the 12 dominant bacterial phyla in the Cerrado biome. Pearson correlation coefficients (r) are shown, with P values corrected using the Benjamini–Hochberg false discovery rate procedure.

We found that the relative abundance of Solibacteres, Acidobacteria subgroup 6, Chloroflexi, Betaproteobacteria, AD3 and Thermoleophilia decreased with higher soil moisture content in the rainy season. In contrast, the relative abundance of Alphaproteobacteria, Gammaproteobacteria, Acidobacteriales, Sphingobacteria, Pedosphaerae, and Gemmatimonadetes increased with higher soil moisture content (Fig 5). Among archaea, only Thermoplasmata was more abundant with increasing soil moisture content; however, this difference was not significant (P> 0.10) (S2 Fig). In fungal communities, the relative abundance of Saccharomyceta and Agaricomycotina differed significantly according to soil moisture content (S2 Fig).

### Functional analysis of microbial communities across vegetation physiognomies and temporal variations of soil moisture

The metabolic potential of the microbial communities was analyzed by MG-RAST, which assigns sequences to metabolic categories based on their best Blastx hit against the SEED database.

**Fig 6:**
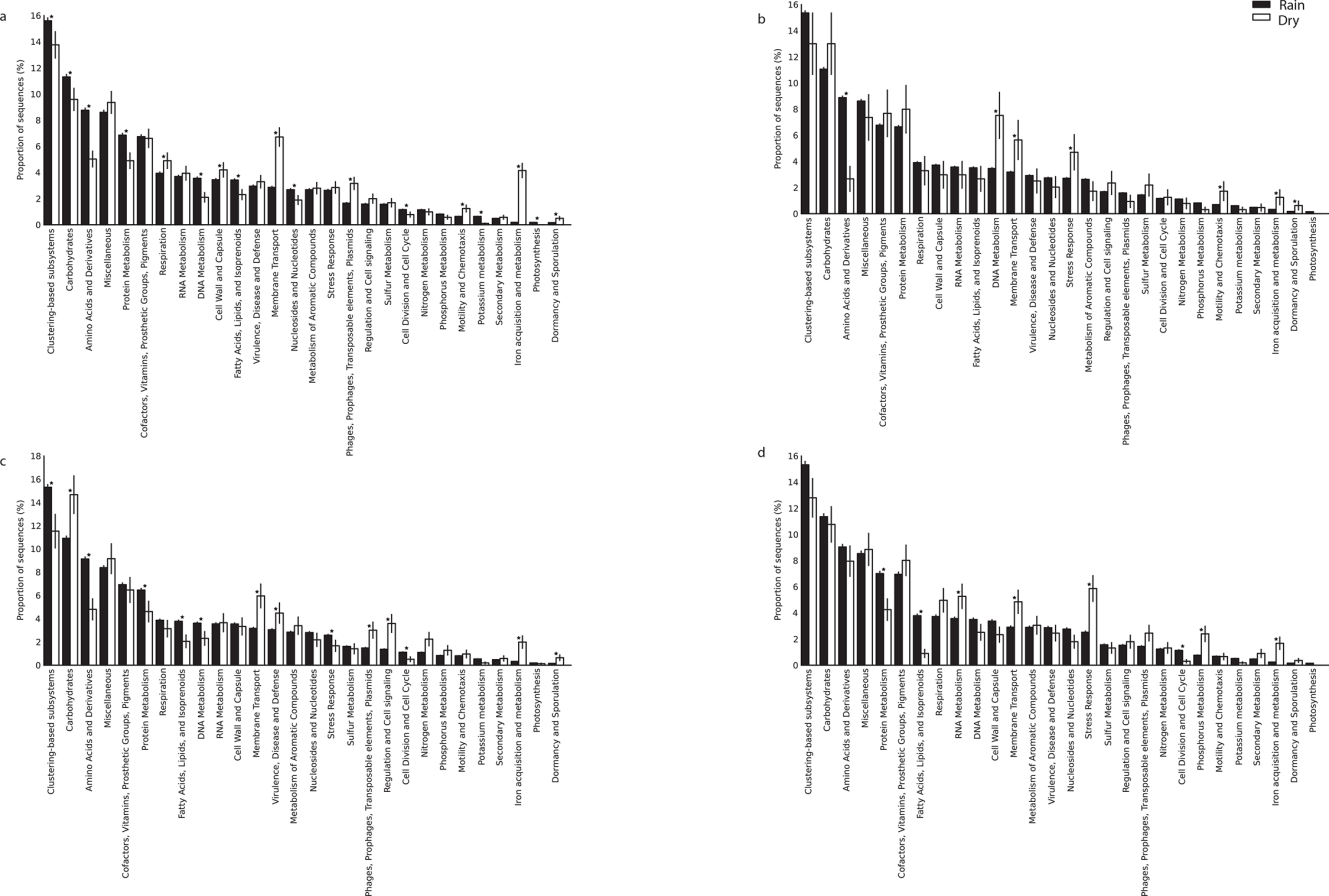
Functional analysis of Cerrado soil metagenomes. The relative abundance of each gene functional category was based on MG-RAST analysis. All data were normalized for the quantity of assigned reads for (a) *cerrado denso*, (b) *campo sujo*, (c) cerrado *sens ustricto*, and (d) gallery forest soils. *P < 0.05 between the dry and rainy season.

Among functional categories, the most frequently encountered genes were assigned to clustering-based subsystems (i.e., functional coupling evidence indicates the genes belong together, but their precise functions are unknown), followed by cell wall and capsule synthesis, dormancy/sporulation, iron acquisition, and assimilation of aromatic compounds (Fig 6). The analysis of genes involved in sulfur and nitrogen cycles indicated that processes associated with nitrogen fixation and sulfide oxidation did not differ significantly between dry and rainy seasons. However, the relative abundance of genes associated with amino acids and their derivatives, DNA/protein metabolism, and cell cycle increased during the rainy season.

## Discussion

### Variation in the abundance of soil microbial communities

In the present study, we demonstrated that soil microbial communities of savanna grasslands (*cerrado denso*, *campo sujo*, and cerrado *sensu stricto*) and forest formations (gallery forest) are profoundly affected by the considerable seasonal variation in water availability that is characteristic of the Cerrado biome. This finding is consistent with other studies reporting the effect of seasonal fluctuations in water availability on the composition and abundance of soil microbial communities worldwide [18, 19].

We combined the description of bacterial, archaeal, and fungal diversity in different Cerrado vegetation physiognomies with metagenome sequencing analysis to explore the potential functional and structural diversity of the Cerrado soil microbial community and demonstrated significant changes in the microbial community associated with water availability. The higher relative abundance of Alphaproteobacteria and Gammaproteobacteria during the rainy season may be due to higher levels of labile carbon and nutrients, as copiotrophic Proteobacteria are associated with soils containing high levels of available carbon contributed by plant litter or root exudation [20, 21]. In contrast, the lower relative abundance of Chloroflexi, Planctomycetes, AD3, and WPS-2 during the rainy season may be due to dormancy induced by the adverse environmental conditions [22]. In some cases, variations in relative abundance of the microorganisms evaluated can be explained by their ecological characteristics. For example, Acidobacteria are known to be tolerant to fluctuations in soil moisture [23, 24], which maybe an important factor for adaptation to the seasonal variations typical of savanna environments. We observed that, in bacterial communities the phylum Acidobacteria was highly responsive to soil moisture. With increasing soil moisture content, members of Acidobacteria subgroup 6 and Solibacteres decreased in relative abundance, whereas members of Acidobacteriales increased in relative abundance. These divergent responses could be due the metabolic versatility of Acidobacteria species, which may facilitate adaptation to environmental changes. Our results are consistent with previous studies evaluating the relative abundance of members of Acidobacteria and the differential response to changes in soil moisture [23, 25]. Although Acidobacteria community composition and interactions have been studied in a variety of environments using cultivation-independent methods, their functions, and in particular their interactions with higher taxa in soil, remain unknown.

Previous studies report that members of Thermoprotei are disproportionately abundant in desert soils, likely because of the radio- and thermotolerant species in this Crenarchaeota class [26]. This observation may explain the higher abundance of Thermoprotei in the *campo sujo* and cerrado *sensu stricto* sites relative to the gallery forest site. *Campo sujo* and cerrado *sensu stricto* are open savanna with scattered shrubs and small trees, which allows greater soil exposure to radiation [27, 28]. Our finding is consistent with that of a previous study, which detected a greater number of bacterial OTUs associated with radiation tolerance in *campo sujo* and cerrado *sensu stricto* soils compared with gallery forest soil [2].

Angel and collaborators (2012) found that methanogens are ubiquitous in soils, including dry land soils, and reported that two specific methanogens, Methanosarcina and Methanocella, appear active when incubated anoxically with water [29]. Similarly, our results show that members of the class Methanomicrobia are more abundant in soil samples of *cerrado denso* with higher water content. In contrast, the class Methanomicrobia was undetected in the dry season, because its relative frequency was lower than 0.1%. Taken together, these results suggest that methanogens are able to survive desiccation stress and will proliferate when water becomes available again.

As expected, Ascomycota and Basidiomycota dominated the fungal communities in our soil samples. The high abundance of Ascomycota has been described in a wide range of soil types, including permafrost soils [30], semiarid grassland soil [31], agricultural and native Cerrado soils [6], and Amazonian rainforest soils [32]. Members of this phylum are able to degrade plant polymers such as cellulose and hemicellulose in woody plant litter [33], which may account for their abundance in a wide range of soils from around the world. In our study the relative abundance of Basidiomycota in gallery forest soil was significantly higher in the dry season; compared with other vegetation types, this physiognomy has a higher proportion of organic matter during the dry season. This finding is consistent with a recent study, which reported that members of Basidiomycota have the ability to decompose complex compounds and tend to colonize soils rich in organic matter [34].

Data from recent studies suggest that the total number of fungal species is much higher than previously thought [35]. In particular, high-throughput DNA sequencing has revealed a surprising number of new fungal species, especially in soils [36, 37]. McGuire and colleagues [38] used high-throughput DNA sequencing to assess fungi in tropical soils, demonstrating that fungal diversity is not associated with plant species richness but strongly correlates with precipitation. Similarly, our results indicate that fungal community structure is influenced by soil gravimetric water content, as demonstrated by β-diversity and Pearson correlation analyses. Previous studies have reported that fungal diversity is more strongly associated with vegetation cover in temperate upland grasslands [39, 40]. However, our results suggest that the precipitation regime may be more important than plant diversity in shaping fungal community structure in tropical savanna soils. These observed seasonal changes in microbial composition associated with water availability are consistent with the results of other studies of tropical forests [25, 41, 42].

In fungal communities the relative abundance of Saccharomyceta and Agaricomycotina negatively correlated with increased moisture content in Cerrado soils, in contrast with the strong positive correlation observed between soil fungal richness and increasing precipitation in neotropical rain forests [38]. Seasonal changes in the fungal community structure have similarly been observed in Alaskan tundra soils [43]. In addition, vegetation cover appears to affect fungal communities in the arctic tundra through nutrient availability, varying root architecture and exudates, and the quality and quantity of litter fluxes [44]. The diversity of fungal communities also correlates with plant community composition in western Amazonian rainforests [32]. This relationship between plant and fungal communities may explain why the relative abundance of Basidiomycota increased in the *campo sujo* and cerrado *sensu stricto* physiognomies during the rainy season, but decreased in the *cerrado denso* and gallery forest physiognomies. Members of the phylum Basidiomycota form a symbiotic relationship with plant roots that enables decomposition of complex lignocellulosic materials [45]. These observations illustrate some of the ways that fungal communities respond to variations in the soil environment.

### Dry and rainy seasons influence the functional microbial diversity

The availability of shotgun metagenome data for Cerrado soils provides an opportunity to compare both data sets. The relative abundance of genes associated with distinct functional categories differed among the four vegetation physiognomies, as well as between seasons, suggesting that microbial communities in these soils are sensitive to both vegetation cover and soil moisture content. Although the factors influencing the incidence and distribution of physiognomic types have not been completely elucidated, Cerrado vegetation physiognomies are known to differ according to nutrient and water availability, aluminum level, and acidity [46]. The biggest difference among the physiognomies analyzed in this study is wood cover [27]. Vegetation influences the soil microclimate by insulating the soil and reducing temperature variability, thereby affecting respiration rate and total carbon balance [47, 48]. However, soil moisture and cover vegetation are not the only factors influencing microbial communities. Changes in pH, temperature, and nutrient availability also influence soil microbial community structure [20, 49, 50]. The relatively higher abundance of DNA and Protein metabolism and Cell cycle genes hints to possible activation of growth of dormant microorganisms. Plants generally represent the largest source of organic carbon in soils, typically rich in lignin-derived aromatic compounds [51]. Therefore, the frequency of genes related to the metabolism of aromatic compounds was expected in these native Cerrado areas, which possess a diverse array of plant species [52]. In contrast, desert soils show a relatively lower abundance of genes related to the metabolism of aromatic compounds [49], probably because they lack a dense vegetation cover.

The well-marked seasons of the Cerrado, which expose microbial communities to frequent moisture stress, may explain the high relative abundance of genes related to cell wall and capsule synthesis, dormancy/sporulation, and iron acquisition. The over representation of genes associated with iron acquisition during the dry season in all four physiognomies may be due to the high iron levels in the soils analyzed in this study. Iron is an essential element for most organisms and can be a limiting factor because of its insolubility at neutral pH in aerobic environments [53]. Compared with the water-saturated soils of the rainy season, soils with low moisture content appear to have higher soil oxygen concentrations [54], which favor bacterial iron oxidation [55]. *Gallionella*-related neutrophilic iron oxidizers (Ga-FeOB) prefer environments with higher O_2_ and Fe^2+^ availability [55]. *Gallionella* was found in the soils of all physiognomies under dry conditions. In the rainy season, genes associated with amino acids and their derivatives, DNA/protein metabolism, and cell cycle increase in relative abundance, indicating that microbial communities proliferate when water is available.

Although we are far from achieving a full understanding of soil microbial communities in the Cerrado biome, our approach combining high-throughput DNA sequencing of rRNA genes with shotgun metagenomics provides insight into some specific functions of soil microbial communities (S3 Fig.) Global climate change has been a growing concern in the last few years, with altered precipitation regimes, increased concentrations of atmospheric CO_2_, and more severe floods and droughts predicted for the future [56, 57]. These precipitation pulses can directly or indirectly influence aboveground communities [58], as seasonal rainfall fluctuations affect the composition and abundance of belowground microbial communities [19, 59]. Several studies have described how the belowground community influences the aboveground community, and vice versa [58, 60]. For example, soil microorganisms can establish mutualistic or antagonistic relationships with plant roots, influencing both the soil microbial community and the macrofauna [61]. However, other interactions between aboveground and belowground components of the ecosystem are not easily predicted. Thus, studies of microbial community structure and functionality in undisturbed soils of Cerrado across different physiognomies and altered precipitation regimes will improve our understanding of key factors determining the remodeling of microbial communities in terrestrial ecosystems.

## Supporting Information

S1 Fig (a) Monthly rainfall (mm) in the IBGE Ecological Reserve, Brasília, DF during the period in which soil samples were collected. (b) Soil moisture measurements in the dry and rainy season in the *cerrado denso* (CD), *campo sujo* (CS), cerrado *sensus tricto* (SS), and gallery forest (MG) (n = 3 for each physiognomy). Error bars represent standard deviations.
(EPS)

S2 Fig Correlations between the relative abundance of the archaea Thermoplasmata and fungi Saccharomyceta and Agaricomycotina and soil moisture content in the Cerrado biome. Pearson correlation coefficients (r) are shown with P values corrected using the Benjamini–Hochberg false discovery rate procedure.
(EPS)

S3 Fig Conceptual map of variation in the relative abundance of soil microbial communities. Main observations from this study as a proposal for structure of soil microbial communities during the well-marked seasons of the Cerrado biome.
(PDF)

S1 Table Physicochemical properties of soils collected from the *cerrado denso*, *campo sujo*, *cerrado sensu* stricto, and gallery forest physiognomies in the dry season. (DOCX)
