# Supplementary figures and images for: Microbial Diversity in Cerrado Biome (Neotropical Savanna) Soils
Source: PLoS One. 2016 Feb 5;11(2):e0148785. doi: 10.1371/journal.pone.0148785 (PMC4743975; doi:10.1371/journal.pone.0148785)

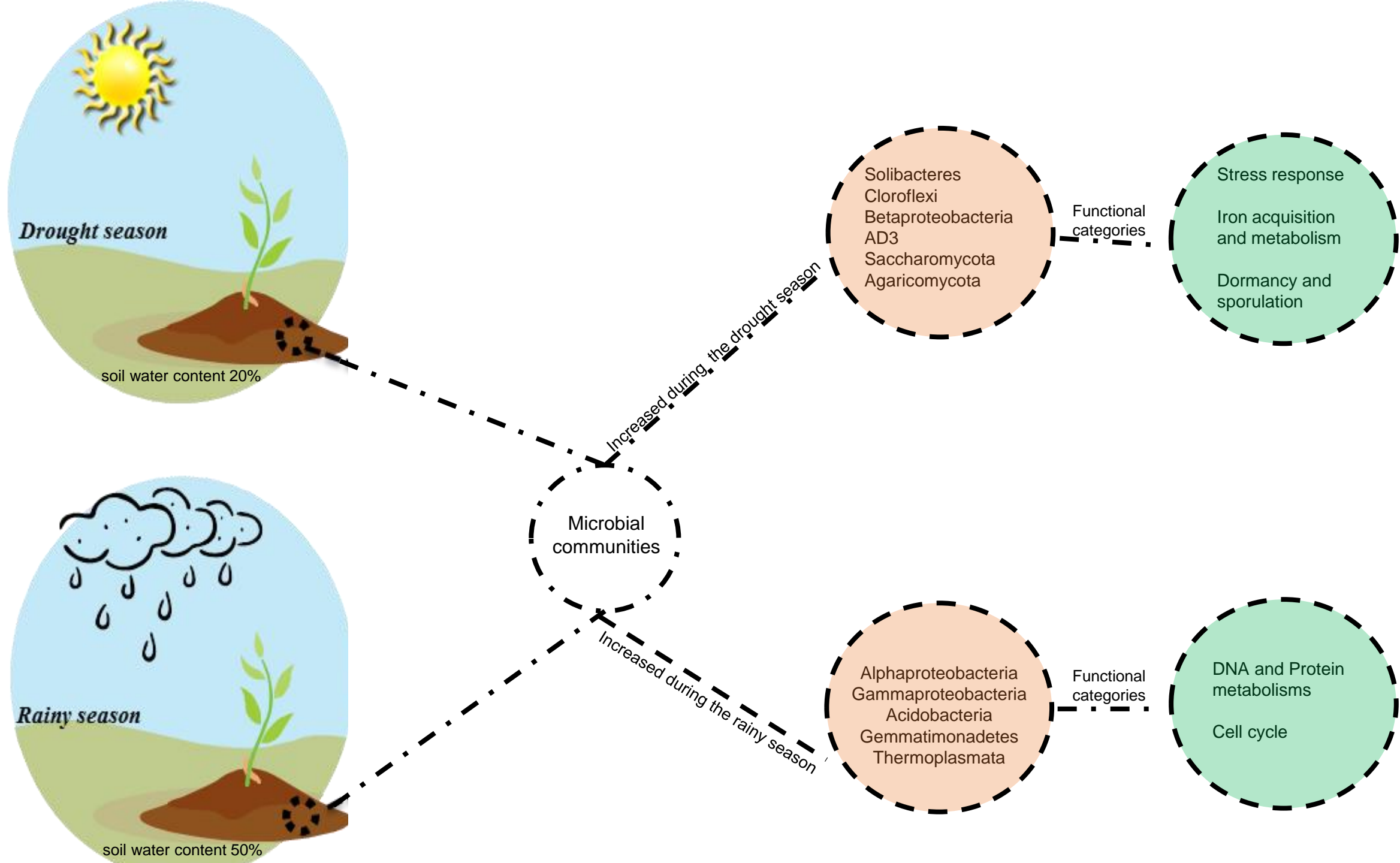

Supplement: S3 Fig — Main observations from this study as a proposal for structure of soil microbial communities during the well-marked seasons of the Cerrado biome. (PDF) [file pone.0148785.s003.pdf]
