# Supplementary material for: Microbial Diversity in Cerrado Biome (Neotropical Savanna) Soils
Source: PLoS One. 2016 Feb 5;11(2):e0148785. doi: 10.1371/journal.pone.0148785 (PMC4743975; doi:10.1371/journal.pone.0148785)
Supplement: S1 Table — (DOCX) [file pone.0148785.s004.docx]

Table S1: Comparison of physicochemical properties from cerrado denso, campo sujo, cerrado *sensu stricto* and gallery forest physiognomy under dry season.

| **Parameters** | **cerrado denso** | **campo sujo** | **cerrado *sensu stricto*** | **gallery forest** |
| --- | --- | --- | --- | --- |
| Clay (%) | 52.5 | 42.5 | 55.0 | 45.0 |
| Sand (%) | 27.5 | 40.0 | 30.0 | 42.5 |
| Silt (%) | 20 | 175 | 150 | 125 |
| pH (H_2_O) | 5.0 | 5.0 | 5.0 | 5.0 |
| P (ppm) | 0.3 | 0.1 | 0.1 | 2.0 |
| Ca (cmolc/dm^3^) | 0.3 | 0.2 | 0.3 | 0.6 |
| Mg (cmolc/dm^3^) | 0.2 | 0.1 | 0.2 | 0.3 |
| K (cmolc/dm^3^) | 0.02 | 0.02 | 0.02 | 0.02 |
| Na (cmolc/dm^3^) | 0.01 | 0.01 | 0.01 | 0.01 |
| Al (cmolc/dm^3^) | 0.9 | 0.9 | 1.9 | 2.2 |
| H + Al (cmolc/dm^3^) | 8.4 | 7.8 | 11.3 | 10.5 |
| Organic carbon (g/kg) | 45.1 | 46.2 | 55.3 | 83.2 |
| Organic matter (g/kg) | 77.6 | 79.5 | 95.1 | 143.1 |
| B (ppm) | 0.65 | 0.25 | 0.1 | 0.63 |
| Cu (ppm) | 0.4 | 0.85 | 0.45 | 0.29 |
| Fe (ppm) | 124 | 36.1 | 70.2 | 124 |
| Mn (ppm) | 7.25 | 65.3 | 26.4 | 19.3 |
| Zn (ppm) | 0.63 | 2.57 | 1.17 | 0.63 |
| S (ppm) | 5.2 | 6.6 | 6.0 | 5.0 |
